# Tractional Descemet’s membrane detachment after ocular alkali burns: case reports and review of literature

**DOI:** 10.1186/s12886-018-0924-x

**Published:** 2018-09-24

**Authors:** Xiaoling Zhang, Vishal Jhanji, Haoyu Chen

**Affiliations:** 1 Joint Shantou International Eye Center, Shantou University & the Chinese University of Hong Kong, Shantou, China; 2 Department of Ophthalmology, University of Pittsburgh, Pittsburgh, USA

**Keywords:** Descemet’s membrane detachment, Ocular alkali burn, Anterior segment optical coherence tomography

## Abstract

**Background:**

Descemet’s membrane detachment (DMD) is a rare complication after ocular chemical injury and its pathogenesis remains unclear. In this study, we reported two cases of DMD with traction demonstrated on Anterior segment optical coherence tomography (AS-OCT).

**Case presentation:**

Two patients sustained ocular chemical injury with 50% sodium hydroxide. In both cases, AS-OCT revealed detached Descemet’s membrane that was adherent to the underlying iris tissue in the inferior quadrant at 45 days and 34 days after the injury respectively. The first case received intracameral tamponade with 12% C_3_F_8_ gas and the second case received corticosteroid and sodium chloride 5% eye drops. However, DMD persisted in both cases.

**Conclusions:**

The atypical features of DMD on anterior segment optical coherence tomography in our cases suggested the presence of an inflammatory component caused adhesions and traction of iris to Descemet’s membrane and prevented reattachment of DMD even with gas tamponade.

## Background

Descemet’s membrane detachment (DMD) is the separation of the Descemet’s membrane from the overlying corneal stroma and is a rare complication of intraocular surgeries (e.g., cataract surgery, surgical iridectomy, etc.) with incidence rates being reported at 2.5% and 0.044–0.5% during extracapsular cataract extraction and phacoemulsification, respectively. [1–6] Chemical injuries to the eye, however, have also been reported as causing DMD and are even rarer. Generally, factors associated with the occurrence of DMD include shallow anterior chamber, use of blunt keratomes, shelved incisions, inadvertent injection of saline, viscoelastic or antibiotics into the supra Descemet’s space and pre-existing endothelial diseases. [7–11] Management of DMD depends on its extent, location, and size. For example, while small, peripheral DMDs can resolve spontaneously, larger more central DMDs require intracameral gas tamponade. If left unattended, DMD may result in the formation of a double anterior chamber, corneal edema and loss of vision.

Descemet’s membrane detachment is a relatively rare complication after ocular chemical injury. Since the initial report by Najjar et al. [12] in 2004, only 3 additional cases of DMD after ocular chemical injury have been reported. [13–15] However, the cases reported in literatures were examined with slit lamp microscopy, which has low resolution. Herein, we describe two cases of DMD after chemical injury with high resolution anterior segment optical coherence tomography (AS-OCT) showing traction from iris which may be the mechanism of DMD in our cases.

## Case presentation

### Case 1

**Fig. 1:**
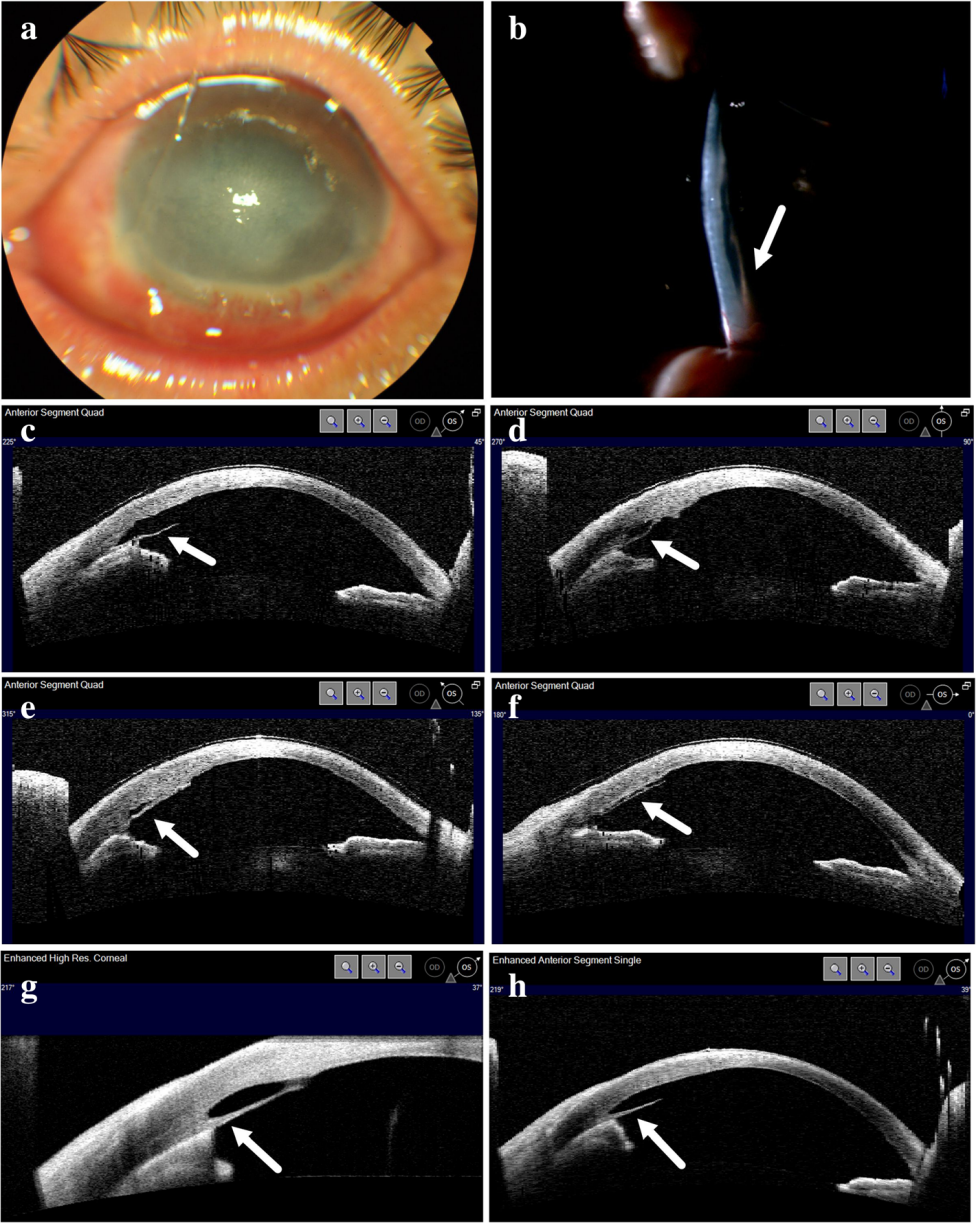
Clinical photographs and anterior segment optical coherence tomography (AS-OCT) of Case 1. **a** Anterior segment photograph of left eye 26 days after injury. **b** Slit lamp photograph 6 weeks after injury showing detached Descemet membrane in inferonasal quadrant. **c**-**f** AS-OCT images at different scan angle at 6 weeks after injury showing detached Descemet’s membrane. **g** AS-OCT images 7 weeks after injury. **h** AS-OCT images 10 weeks after injury

A 44-year-old male was referred to our hospital 26 days after an accidental chemical injury in his left eye with 50% sodium hydroxide solution. The patient irrigated his left eye with tap water immediately after the injury and was subsequently treated at a local clinic. At the time of presentation to our hospital, his visual acuity was hand movements in the left eye and 20/20 in the right eye. Slit-lamp examination revealed an inferior corneal epithelial defect involving 2 clock hours along with diffuse corneal epithelial and stromal edema (Fig. 1a). The patient was treated with 0.3% ofloxacin four times a day, pranoprofen 1% four times a day, 0.1% prednisolone acetate eye drops four times a day, 1% atropine sulfate eye gel twice daily and oral 2000 mg vitamin C per day. The patient was advised to follow-up in our clinic on a weekly basis. The epithelial defect resolved at one week follow up, when the best-corrected visual acuity (BCVA) improved to 20/400. Six weeks after the initial injury, DMD was noted in the inferonasal quadrant on slit-lamp examination (Fig. 1b) and the BCVA was 20/200. AS-OCT scan confirmed a localized DMD in the inferior quadrant. The detached Descemet membrane was thick and adherent to the underlying iris tissue. The iris was pulled anteriorly (Fig. 1c-f). Confocal scanning microscopy failed to detect the corneal endothelium. On the following day, 0.1 ml of 12% perfluoropropane (C_3_F_8_) gas was injected into the anterior chamber. However, the detached Descemet’s membrane persisted postoperatively (Fig. 1g and h). The central cornea clarity improved gradually and the BCVA of the left eye recovered to 20/100 and 20/50 at two and four months after the initial injury.

### Case 2

**Fig. 2:**
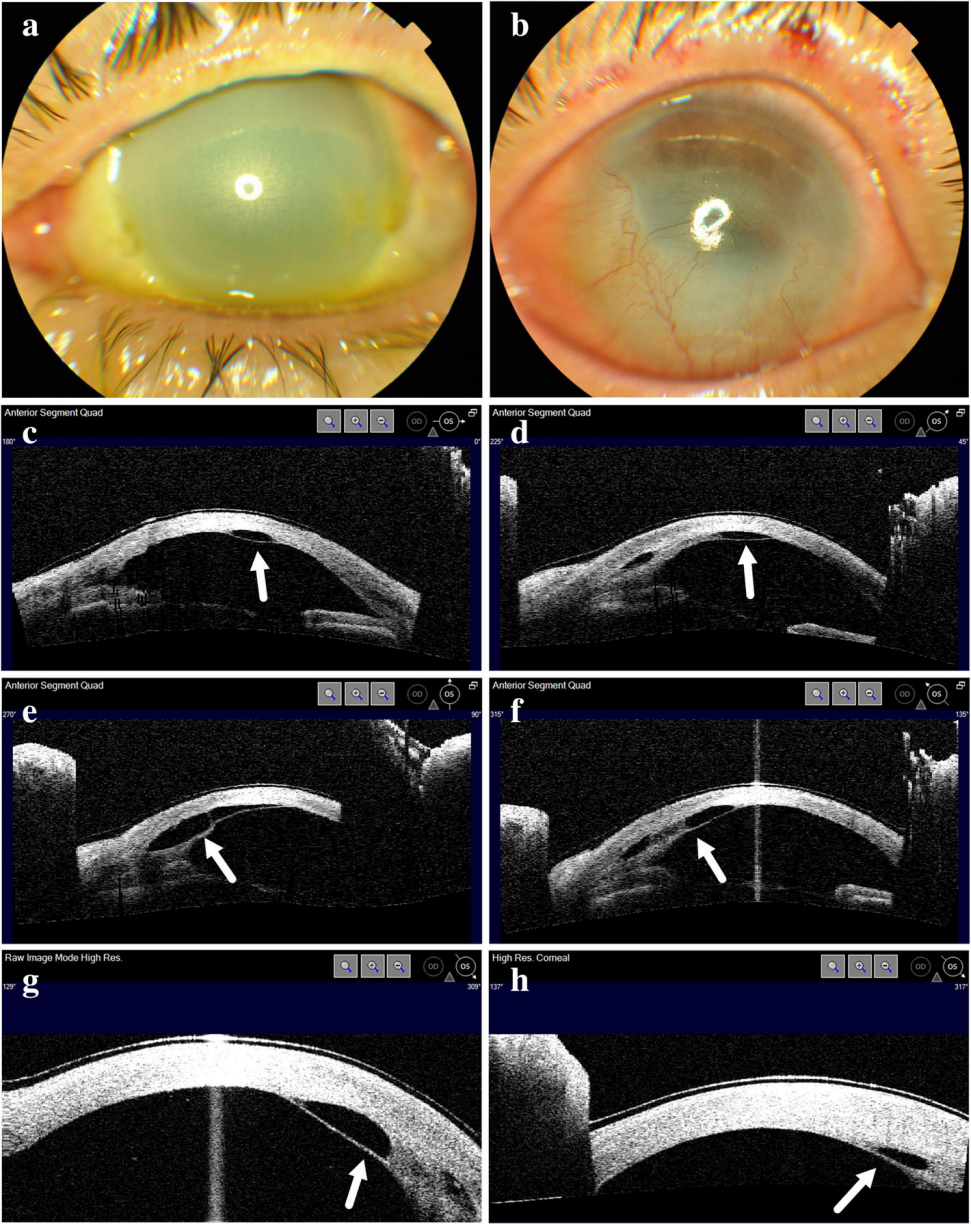
Clinical photographs and anterior segment optical coherence tomography (AS-OCT) of Case 2. **a** Anterior segment photography of left eye 1 day after injury. **b** Anterior segment photography of left eye 1 year after injury showing severe corneal neovascularization. **c**-**f** AS-OCT images at different scan angle at 5 weeks after injury showing detached Descemet’s membrane at the inferior part. **g** AS-OCT images 6 weeks after injury. **h** AS-OCT images 9 weeks after injury

A 28-year-old male presented to our hospital after ocular chemical injury with 50% sodium hydroxide. The patient irrigated his eyes with tap water immediately after the injury and presented an hour later to our hospital. At the time of presentation, the BCVA was 20/40 in the right eye and 20/200 in the left eye. There was extensive corneal edema along with limbal ischaemia (Fig. 2a). Treatment was commenced in the form of topical levofloxacin 0.5% four times a day, topical pranoprofen 1% four times a day, topical atropine 1% gel nocte and oral vitamin C 2000 mg per day. Five weeks later, slit lamp examination revealed a DMD, which had not been present during the initial examination. AS-OCT showed a localized DMD in the inferior quadrant. Similar to the first case, the detached Descemet membrane was thick, adherent to the underlying iris tissue and pulled the iris anteriorly (Fig. 2c-f). Topical prednisolone acetate 1% eye drops and sodium chloride 5% eye drops were administered every 2 h; however, the DMD persisted at the end of one week and one month (Fig. 2g and h). There was severe corneal neovascularization (Fig. 2b) and the BCVA of left eye dropped to hand movements at one-year follow-up visit. He received penetrating keratoplasty at another institute and the BCVA improved to 20/200 postoperatively.

## Discussion and conclusions

In iatrogenic cases, DMD may arise as a result of direct contact between the corneal endothelium and surgical instruments. [7–11] In cases with chemical injuries, the cause of DMD remains unclear, which may due to limited cases reported in literature. Table 1 summarize the characters and proposed mechanism of the cases reported in literature and current article.

**Table 1:** Summary of cases of Descemet’s membrane detachment after ocular chemical injuries in literature and our study

Article	Yuen HK 2004	Zhang B 2012	Najjar DM 2004	Najjar DM 2004	Hua MT 2010	Case 1 in our report	Case 2 in our report
Age	40	19	49	45	26	44	28
Gender	Male	Male	Male	Female	Male	Male	Male
Chemical	hydrogen peroxide	sodium cyanide	sodium hydroxide	unknown	ammonia	sodium hydroxide	sodium hydroxide
Onset	3 days	4 days	4 months	4 months	2 months	6 weeks	5 weeks
Location	inferior	Extensive	inferior	inferior	Inferior	inferonasal	inferior
Exam	Slit lamp	UBM	Slit lamp	Slit lamp	Slit lamp	AS-OCT	AS-OCT
Initial VA	HM	20/800	20/80	20/800	HM	HM	20/200
Hyphema	No	No	Yes	Yes	Yes	No	No
Management	Intracameral 20% SF_6_ injection	1% prednisolone and 0.5% levofloxacin eye drops	Intracameral 18% SF_6_ injection	unknown	Intracameral air bubble injection	Intracameral 12% C_3_F_8_ Intracameral	1% prednisolone and 5% NaCl eye drops
Outcome	reattached	reattached	unresponsive	unknown	unknown	unresponsive	unresponsive
Final VA	20/30	20/100	20/400	unknown	unknown	20/50	HM
Proposed mechanism	hydrogen peroxide penetrated and formed gas anterior to DM	Severe cellular damage in the stroma and endothelial layer	1) an inflammatory retrocorneal membrane associated with an organizing hyphema that pulled on DM leading to its detachment.2) retrocorneal membrane develop neovascularization that rupture and fill the space between DM and stroma		Not proposed	contraction of fibrous exudates pulls on the iris and DM together to its detachment	
Associated evidences	gas bubble between corneal stroma and Descemet membrane.	no normal keratocytes /endothelial cells could be detected by in vivo confocal microscopy	a thickened, detached DM associated with a hyphema		Accompanied with hyphema	AS-OCT showed that the detached DM was thick and adherent to the iris. There is no hyphema	

*VA* visual acuity; *HM* hand movement; *DM* Descemet’s membrane; *AS-OCT* anterior segment optical coherence tomography

There are two cases developed DMD at 3–4 days after the chemical injury, and reattachment were achieved spontaneously or with 20% SF_6_ injection. [14, 15] In these cases, the detached Descemet membrane was loose on slit lamp examination. Yuen et al. [14] observed gas bubble between corneal stroma and Descemet membrane, so they suggested that chemicals like hydrogen peroxide may generate gas, which pushes the Descemet membrane thereby detaching it from the overlying corneal stroma. While Zhang et al. [15] found no normal keratocytes /endothelial cells could be detected by in vivo confocal microscopy and proposed cellular damage of stroma count for DMD.

In the cases reported by Najjar et al. [12], Hua et al. [13], DMD occurred 1–4 months after the initial injury. The attempt to reattach the Descemet’s membrane were unclear or failed in these cases. These cases had associated signs of anterior chamber inflammation and hyphema. Najjar et al. [12] hypothesized two possible mechanisms: 1) an inflammatory retrocorneal membrane with an organizing hyphema that pulled on to the Descemet membrane leading to its detachment; 2) development and subsequent rupture of neovascularization on the retrocorneal membrane leading to collection of blood between the corneal stroma and Descemet’s membrane.

**Fig. 3:**
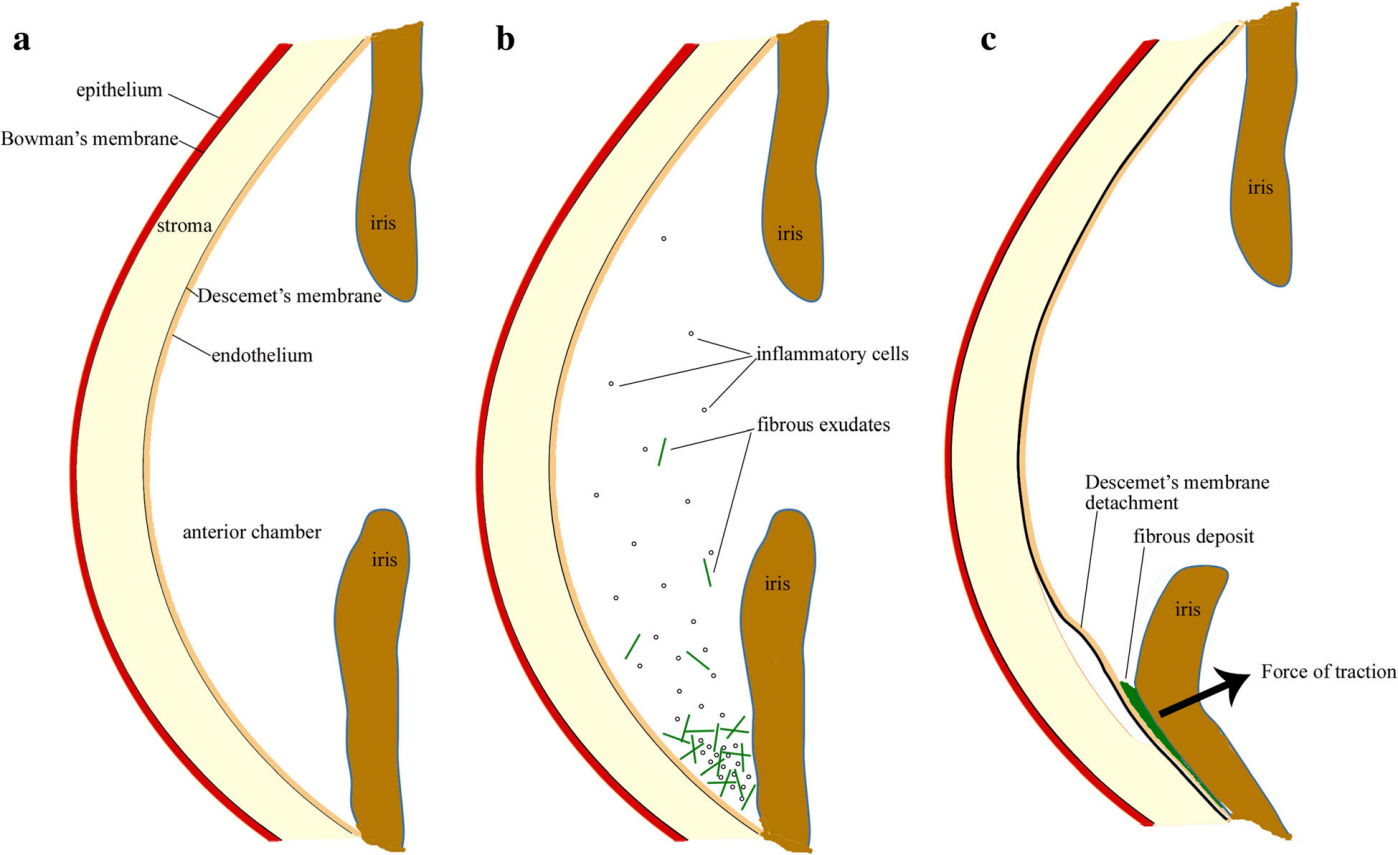
Diagram of cross-section of the anterior segment. **a** Normally the anterior chamber is quiet and there is an angle between cornea and iris. **b** In the eyes with chemical injury, there are inflammatory cells and fibrous exudates in anterior chamber. **c** The fibrous exudates gravitate down in the anterior chamber angle, causing iris adhesions to the Descemet’s membrane. Contraction of the iris causes Descemet’s membrane detachment

Our cases also developed DMD more than 1 month after chemical injury, similar to the cases reported by Najjar et al. [12], Hua et al. [13]. However, no hyphema was noted in our cases. AS-OCT showed that the detached Descemet’s membrane was thick and adherent to the iris. Based on the clinical presentation and AS-OCT features, we hypothesize that the following pathogenesis: chemical injury induced inflammation in anterior chamber; the inflammatory cells and fibrinous exudates gravitate down in the inferior anterior chamber causing adhesions between the iris and Descemet’s membrane; contraction of the fibrinous exudates and iris caused detachment of Descemet’s membrane (Fig. 3). Although we did not have access to the tissue to investigate the histopathological characterization, high resolution AS-OCT provided cross-sectional images and clearly demonstrated the relationship between Descemet’s membrane and the underlying tissue which provides supporting evidence for our hypothesis.

The management options for DMDs include conservative treatment, pneumodescemetopexy, suture fixation and even keratoplasty. To date, no prospective studies on the choice of management of DMDs have been published. [16] The reference of management for DMD after chemical injury is more limited due to small number of cases published. Resolution of post-chemical injury DMD has been reported in early onset cases with 20% SF_6_ intracameral injection or even spontaneously. The DMD was loose in these cases. The DMD failed to reattach even after intracameral gas tamponade in late onset cases, including Najjar et al.’s case and our first case. This may due to traction force of the underlying iris tissue. In the second case with poor visual acuity, keratoplasty is needed.

In conclusion, DMD is an uncommonly reported complication after ocular chemical injury. The atypical features of DMD on AS-OCT in our cases suggested the presence of an inflammatory component caused adhesions and traction of iris to Descemet’s membrane and prevented reattachment of DMD even with gas tamponade. Further studies with larger sample size is needed to confirm the pathogenesis proposed in this study and to explore therapy for these cases.

## Abbreviations

AS-OCT: Anterior segment optical coherence tomography
C_3_F_8_: Perfluoropropane
DMD: Descemet’s membrane detachment
